# Status of COPD in Saudi Arabia: challenges, gaps, and opportunities for health system transformation

**DOI:** 10.3389/fpubh.2026.1904870

**Published:** 2026-07-29

**Authors:** Jaber S. Alqahtani

**Affiliations:** Department of Respiratory Care, Prince Sultan Military College of Health Sciences, Dammam, Saudi Arabia

**Keywords:** Burden, COPD–chronic obstructive pulmonary disease, digital health, health system transformation, pulmonary rehabilitation (PR), respiratory care (RC), Saudi Arabia, smoking

## Abstract

Chronic obstructive pulmonary disease (COPD) is a serious public health problem and unfortunately, policies and health system structures in Saudi Arabia remain inadequate to address its rising healthcare burden. This review aims to map and critically analyze the existing evidence on COPD management in Saudi Arabia and to propose a framework to guide clinical practice and improve patient outcomes. Globally and particularly in Saudi Arabia, COPD remains significantly underdiagnosed despite the availability of well-established clinical guidelines. Based on the current analysis, this is primarily due to a lack of public awareness, inconsistent application of spirometry in primary care settings, and delayed patient presentation. Notable policy deficiencies remain, including the lack of a unified national COPD strategy, inadequate implementation of tobacco control measures, and the absence of established protocols for long-term treatment, pulmonary rehabilitation, and home-based respiratory care. Challenges within the health system exacerbate these disparities, including inconsistent access to pulmonary rehabilitation, a shortage of specialist respiratory care providers, disjointed digital health integration, and inconsistent coverage for home oxygen and sophisticated respiratory equipment. The absence of national COPD registries and the lack of dedicated funding for COPD-related projects hinder effective monitoring and delay data-driven planning and leadership. For better COPD outcomes, it is essential to implement coordinated national policies, strengthen workforce capacity, and expand rehabilitative and community-based programs. These efforts should be supported by a robust digital and data infrastructure to ensure integrated, high-quality COPD care across the Kingdom.

## 1 Introduction

Chronic Obstructive Pulmonary Disease (COPD) is a systemic, heterogeneous condition characterized by abnormalities of the airways and/or alveoli, resulting in airflow obstruction and persistent chronic respiratory symptoms (1). COPD affects more than 400 million individuals globally, positioning it as the third leading cause of mortality worldwide (2). People living with COPD frequently present with comorbidities, especially cardiovascular, renal, and mental diseases, which are associated with poor health outcomes (3, 4). The economic and social implications of COPD are significant, with projections indicating that it will generate over $4 trillion in direct and indirect costs annually from 2020 to 2050 (5). Conversely, expenditures aimed at mitigating the burden of COPD appear to be disproportionately low, as evidenced by the allocation of only 0.01% of Europe's largest health program budget for the years 2021–2027 to chronic respiratory diseases (6). In addition to the apparent financial toll on healthcare systems, COPD has far-reaching effects on economic activity due to the high rates of hospitalization, disability, and early retirement experienced by people living with COPD (2). These factors cause them to spend less time working, which, in turn, places a burden on families and the economy as a whole (2).

In Saudi Arabia, the burden of COPD is significant, with approximately 435,000 individuals affected in 2019, resulting in a prevalence rate of 2,053.04 cases per 100,000 population (around 2.05%) (7). Compared with other Middle Eastern countries, Saudi Arabia ranks among those with the largest increases in COPD incidence, with an estimated annual percentage change of approximately 1.17 from 1990 to 2019 (8). Despite this high burden, a nationwide awareness survey indicates a significantly low level of public awareness, with approximately 77% of respondents having never heard of COPD (9). Additionally, there is a low uptake of pulmonary function tests (PFTs) across various regions, highlighting disparities in both knowledge and access to diagnostics (9). Inadequate care management, limited adherence to guidelines, and labor shortages are additional factors that influence the delivery of COPD care (9–11). In response to this pressing need in Saudi Arabia, the main stakeholders in healthcare delivery, including policymakers, healthcare providers, and patients, should collaborate to increase public awareness of COPD and identify the most effective strategies for preventing the condition and improving health outcomes for individuals living with it. This state-of-the-art narrative review aims to identify the unmet healthcare needs associated with COPD management in Saudi Arabia, thereby underscoring the urgent need for a holistic approach from policy to practice to enhance COPD care.

## 2 Methods

This narrative review synthesizes the current status of COPD in Saudi Arabia from heterogeneous epidemiological, clinical, and policy evidence. A structured search was conducted from inception until April 2026 across PubMed/MEDLINE, Scopus, EMBASE, and Google Scholar, supplemented by reference lists, and reports from the Saudi Ministry of Health, WHO, GOLD, and the Global Burden of Disease projects. Controlled vocabulary and free-text keywords were combined using Boolean operators and truncation around the condition (“chronic obstructive pulmonary disease” OR COPD OR “chronic bronchitis” OR emphysema), setting (“Saudi Arabia” OR Saudi OR KSA OR Gulf OR “Middle East”), and thematic terms (prevalence, burden, mortality, underdiagnosis, spirometry, diagnosis, management, smoking, waterpipe, risk factors, health system, primary care). Papers were screened by title, abstract, and full text, with selection guided by thematic relevance; priority was given to primary Saudi data, national surveys, and official statistics, while Gulf and Middle Eastern evidence was used and explicitly identified where Saudi data were sparse. Findings were synthesized narratively and organized thematically into two main themes: the regulatory and policy landscape, and access and care coverage for COPD. This aims to identify diagnostic and management gaps, health-system constraints, and opportunities for future transformation.

## 3 Regulatory and policy landscape for COPD

### 3.1 National COPD strategy

Having a COPD national strategy in each country is paramount (12). A strategy for COPD is defined as a holistic approach that involves prevention strategies, population-based surveillance programs, evidence-based interventions, and early detection initiatives at a population scale (12, 13). Studies conducted globally demonstrate that countries that establish official national strategies achieve greater diagnostic precision, better treatment adherence, and improved patient outcomes at lower healthcare costs and shorter hospital stays (12). In Saudi Arabia, there is no standalone national COPD strategy to mitigate the high burden of COPD on society.

Through an effective COPD strategy, clinical guidelines can be integrated with public health strategies and workforce capacity and resource allocation to create a unified approach to COPD management, replacing independent, separate initiatives (14). Such a strategy can lay the groundwork for essential service routes, including spirometry-based early diagnosis, treatment procedures aligned with guidelines, access to pulmonary rehabilitation, and organized follow-up care (13, 15). The presence of national frameworks in countries leads to better performance results. According to available evidence, implementing systematic approaches leads to better diagnostic outcomes, increased referrals for pulmonary rehabilitation, and improved compliance with evidence-based guidelines (16, 17). Studies in England, Germany, Canada, and Japan have demonstrated that unified COPD treatment approaches result in financial benefits and longer periods of quality-adjusted life expectancy (13).

The Speak Up for COPD coalition released a national profile for Saudi Arabia that calls for enhanced policy focus and the full integration of COPD into national health planning strategies (12). Therefore, it's vital to establish a national COPD strategy in Saudi Arabia that aligns with Vision 2030 goals. I therefore propose a national framework for COPD improvement in Saudi Arabia.

This proposed framework includes the following core national strategies under the national acronym SCOPE: Saudi COPD Optimization and Policy Enhancement:

• **Saudi population engagement and awareness**

Enable and empower the public, people living with COPD, their families and caregivers, and healthcare providers through awareness, education, digital tools, and community support to identify and mitigate the impact of COPD.

• **Care quality enhancement**

Enhance the quality of care provided throughout the healthcare continuum to improve the prevention, early diagnosis, treatment, and management of COPD.

• **Outcomes and data surveillance**

Collect, evaluate, record, and share public health data on COPD through national data registries to drive change and monitor progress.

• **Promotion of fund and research activity**

Enhance funding and maintain research activity to improve our understanding of the prevention, etiology, diagnosis, treatment, and management of COPD.

• **Evidence-based recommendations implementation**

Translate national guidelines, educational initiatives, and program recommendations into actionable public health interventions.

**Figure 1:**
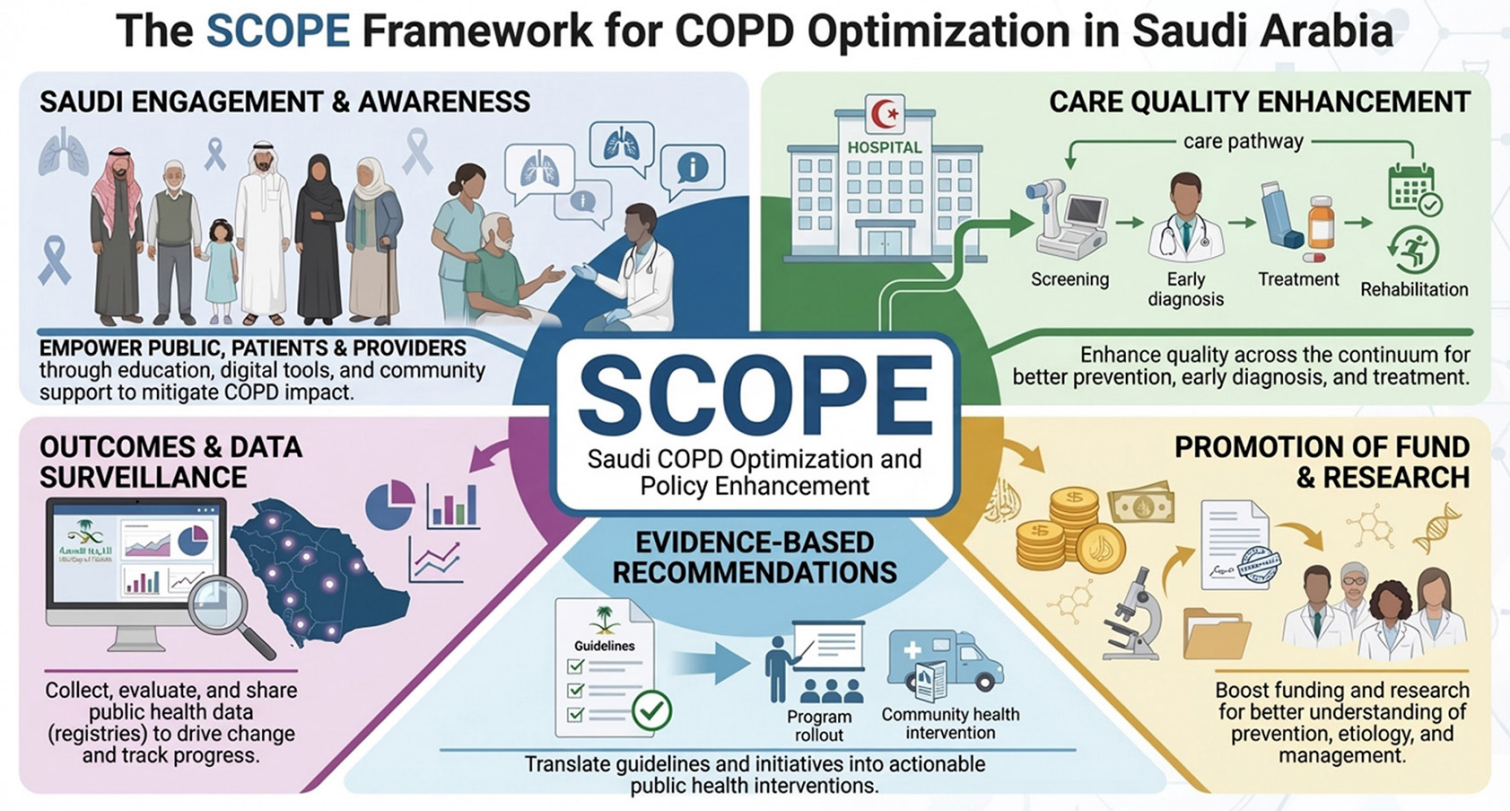
The SCOPE Framework for COPD Optimization in Saudi Arabia.

Saudi Arabia, with its strong government and forward-thinking Vision 2030, is well-positioned to lead regional innovations in respiratory health. A tailored plan for COPD would represent a significant step forward in achieving that vision (Figure 1).

### 3.2 Guidelines for COPD care

Numerous national and international societies and organizations have published guidelines for COPD in response to the growing burden of the disease (18). Where guidance is available, national COPD guidelines are frequently based on the GOLD report (1). However, it is crucial to have specific guidelines tailored to the needs of each country or region. The diagnosis, management, and prevention of COPD in any country can be significantly enhanced by the availability of evidence-based guidance, further strengthened by effective dissemination and implementation strategies (18). In Saudi Arabia, the first COPD guidelines were developed in 2008 to lessen the burden of COPD and help mitigate its risk. Subsequently, an update was implemented in 2014, prompted by advancements in this domain that demanded a revision of the guidelines (19). To achieve Saudi Vision 2030, the Saudi Ministry of Health (MOH) has established a national guidelines program to ensure evidence-based clinical treatment for all illnesses, including COPD. In 2025, the Saudi Thoracic Society and MOH worked together to produce an updated version of the COPD guidelines (20). However, the steering committee responsible for creating this work lacked diversity and was not multidisciplinary; it mostly consisted of physicians and excluded other crucial health care providers involved in the care of COPD patients, such as respiratory therapists, nurses, family medicine providers, PTs, and pharmacists.

Excluding multidisciplinary teams from the development of national COPD guidelines may result in recommendations that are limited, clinically insufficient, and poorly suited to real-world practice. In the absence of contributions from primary health care providers, public health experts, and patient advocates, guidelines may fail to address critical implementation barriers, including access to diagnostics, continuity of care, and cultural relevance. In Saudi Arabia, this may lead to fragmented management of COPD, hindering the effectiveness of national initiatives aimed at improving outcomes and aligning with Vision 2030 goals.

In summary, despite noted limitations in developing national COPD guidelines, Saudi Arabia has made significant progress in standardizing COPD care compared to other countries. Still, comprehensive system-level reforms are essential to turn these guidelines into greater patient outcomes.

### 3.3 Occupational exposure policy

The government of Saudi Arabia has implemented various national laws and sector-specific policies to control occupational exposure in industries that face high risks of chemical and airborne pollutants. The Law of Management of Chemical Substances and the Occupational Safety and Health (OSH) Standards issued by the Ministry of Human Resources and Social Development have provided a framework for monitoring workplace hazards, including respiratory exposures (21). However, different high-risk business sectors lack standardization in their surveillance and enforcement practices. The 2021 GCC Unified Guidelines for Hazardous Chemical Substances Management, developed with the assistance of the United Nations Environment Program, provide a unified regional system that supports Saudi Arabia's initiatives through standardized classification and labeling, as well as safe handling procedures (22). A recent systematic review found that exposure to chemical hazards in Saudi Arabia, including ammonia, pesticides, industrial fumes, and incense smoke, has resulted in an increased respiratory burden, biochemical changes, and decreased lung function in various work environments (23). Thus, organizations need to establish more effective workplace safety rules to minimize the development of COPD and the associated persistent health issues. Implementing such guidelines in national occupational health plans will reduce long-term exposure to airborne pollutants in the industrial, energy, and construction sectors, thereby lowering the COPD burden and contributing to achieving Vision 200′s goals for preventive health and environmental safety.

### 3.4 Tobacco control policy

The government of Saudi Arabia has successfully implemented tobacco control measures that follow the recommendations of the WHO Framework Convention on Tobacco Control (FCTC) and the MPOWER package (24). The WHO Report on the Global Tobacco Epidemic 2023 indicates that Saudi Arabia meets all the requirements of the FCTC in several areas, including the ban on tobacco advertising, promotion, and sponsorship, which encompasses all forms of advertising (25). The report also displays that the total tax share on cigarettes reached 73.8% and that cigarettes have become less affordable since 2012, reflecting robust financial control under the “R—Raise taxes” measure (25). The Saudi Arabian government has adopted plain packaging for tobacco products and requires health warning labels that include details of a smoking cessation hotline, thus demonstrating strong adherence to the “W—Warn about the dangers of tobacco” recommendation (25). However, the country's anti-tobacco measures in mass media are notably minimal. Further, the government operates a national quit line. It offers nicotine replacement therapy as part of its comprehensive cessation framework, making it one of the few countries in the Eastern Mediterranean Region that meet the WHO “O—Offer help to quit” criteria (24).

Nevertheless, the report highlights a moderate level of adherence to the principle of safeguarding individuals from tobacco smoke, as the implementation of smoke-free environments in public and workplace contexts remains insufficient (25). Regarding “M—Monitor tobacco use and prevention policies,” Saudi Arabia has moderate adherence to WHO standards for recent, representative data for both adults and adolescents, but not for periodic surveillance data (25). Overall, Saudi Arabia's tobacco control strategy features a robust legislative framework, an effective taxation system, and cessation services; however, it requires enhanced monitoring and enforcement to ensure comprehensive protection of its population from tobacco exposure.

## 4 Access and care coverage for COPD

### 4.1 Treatment and drug access

The availability of treatment and drug coverage for COPD in Saudi Arabia has shown significant improvement over the last few years, although the healthcare system continues to face ongoing difficulties in providing equal-standard, guideline-based care across all areas and medical facilities. The Saudi Ministry of Health works together with other national societies to improve drug availability, while implementing standardized care procedures that follow international best practices for COPD pharmacological management (20). The Council of Health Insurance (CHI) functions as a core element of Saudi Arabia's medication equity system that enhances COPD care accessibility across the country (26). Within Saudi Arabia's unified health insurance framework, the CHI Drug Formulary establishes a standardized national list of approved medications that all insured individuals, including those in the private sector, are entitled to receive under their health insurance plans (26). The formulary provides patients with necessary COPD medications, including bronchodilators, inhaled corticosteroids, and combination inhalers, which follow both Saudi and international treatment protocols for COPD (27). Irrespective of patients' income or geography, access to treatment must be available to all COPD patients in accordance with CHI regulations. This mandate helps to eliminate previous treatment access disparities that mainly affected COPD patients throughout Saudi Arabia's developing healthcare network (20, 27). Indeed, there is a lack of research regarding the economic burden of COPD on patients and healthcare systems in Saudi Arabia, necessitating further investigations. This burden encompasses both direct healthcare costs, such as hospitalizations and medications, and indirect costs, such as lost income and productivity.

### 4.2 Diagnosis and spirometry access

Recent data from the Global Burden of Diseases, Injuries, and Risk Factors Study (GBD) 2023 indicate that COPD is the third leading cause of death worldwide (28). Saudi Arabia is seeing a substantial rise in the burden of COPD, with over 434,560 individuals affected in 2019, indicating a 49% increase in age-standardized prevalence since 1990 (7). The significant disease burden contrasts with diagnostic rates, highlighting extensive underdiagnoses among the community. The Saudi clinical guidelines, along with the GOLD report, use spirometry as the primary diagnostic tool for COPD, underscoring its importance in diagnosis and management (1, 20). The majority of urban public hospitals, along with specialized pulmonary clinics, maintain spirometry equipment and qualified staff to perform the test, thus providing easy access to the test for numerous patients.

However, several studies reveal underutilization and access gaps in primary health care facilities throughout the Kingdom (9, 10, 19, 29). A recent meta-analysis indicates that the prevalence of undiagnosed COPD among symptomatic smokers in primary healthcare settings is approximately 14–26% (30). Undiagnosed COPD is evident and common in the daily practices in Saudi Arabia, where diagnostic tools remain limited (31). The available Saudi data on urban-rural access to spirometry is limited. Yet, the high number of specialized services in major urban areas creates significant access challenges for people living in rural and remote areas. The lack of spirometry availability in primary care facilities poses a significant problem, as rural areas often rely on these facilities as their primary point of access to healthcare (32, 33). In Saudi Arabia, the level of education proves to be a significant factor that affects people's awareness of COPD and their willingness to seek medical care (9).

**Figure 2:**
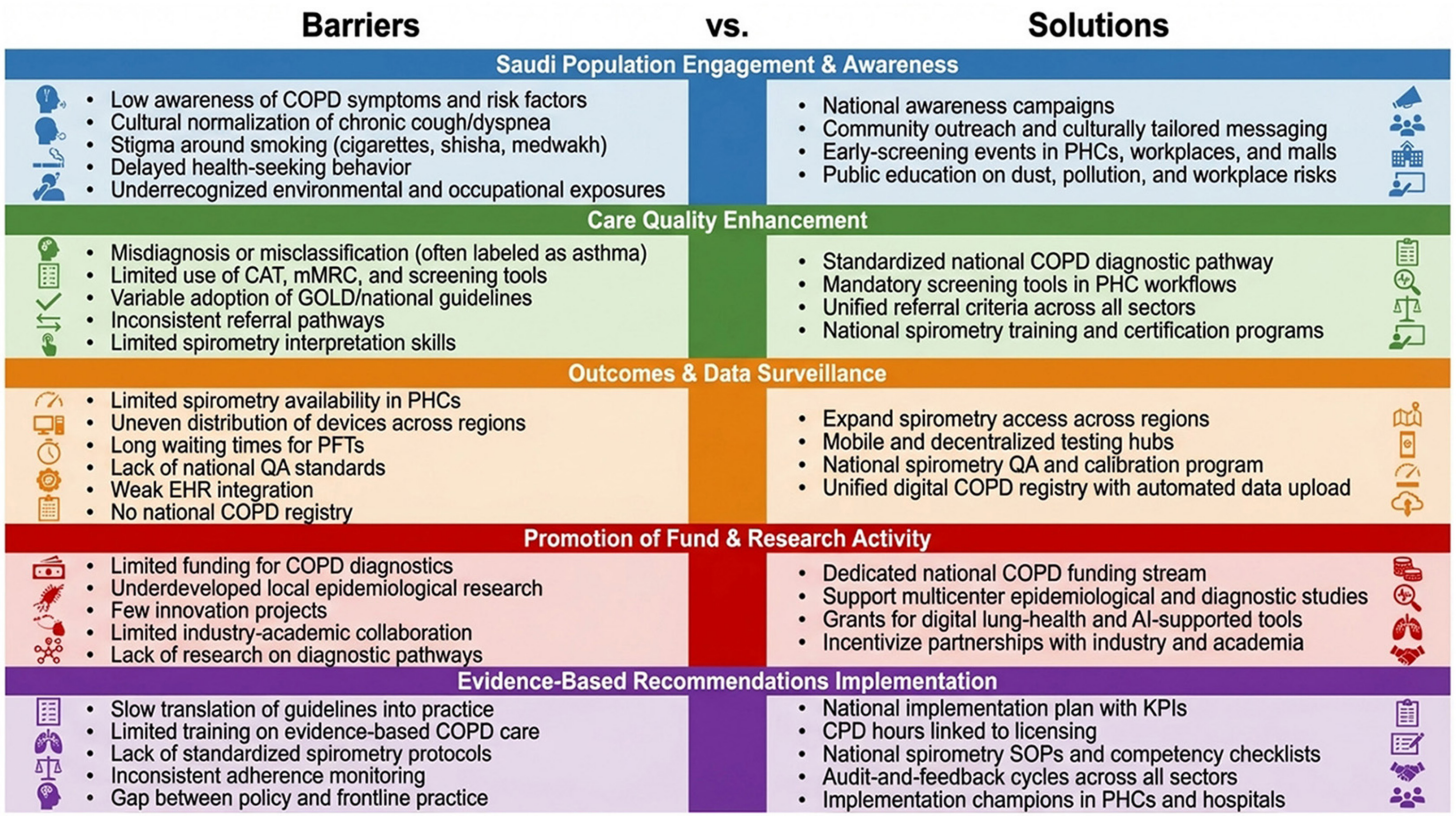
Barriers and corresponding solutions for COPD diagnosis and spirometry access in Saudi Arabia.

Additionally, healthcare professionals involved in COPD management often have limited knowledge and training, which can lead to suboptimal care (9–11, 34, 35). The observed pattern suggests that socioeconomic differences may lead to varying diagnostic rates across population groups. Figure 2 shows the barriers and corresponding solutions for COPD diagnosis and spirometry access in Saudi Arabia, aligned with the proposed SCOPE framework.

### 4.3 Pulmonary rehabilitation and ambulatory/home oxygen access

Pulmonary rehabilitation (PR) and ambulatory/home oxygen are vital non-pharmacological elements of COPD management; however, their availability is inconsistent across Saudi Arabia (20, 36). This uneven access is evident despite explicit guideline recommendations and increasing government focus on these programs (20).

#### 4.3.1 Pulmonary rehabilitation access

The latest Saudi Thoracic Society COPD guidelines strongly advocate PR for patients with stable COPD, particularly recommending referral to structured PR programs for those with a higher symptom burden (20). Evidence from reviews centered on Saudi Arabia indicates that PR enhances exercise capacity, reduces dyspnea, and improves quality of life, while also being cost-effective relative to pharmacological treatment alone, consistent with international findings (36, 37). In 2019, Saudi Arabia's healthcare expenditure was approximately SR 93.7 billion, and this increased to 105 SR billion in 2023 (38). Reducing health care expenditure requires the development of effective strategies, including PR, to alleviate the burden of chronic diseases such as COPD (7). Although the first PR program in Saudi Arabia was established at King Abdulaziz Medical City in 2001, access to PR remains limited or entirely unavailable in numerous regions, especially beyond major urban centers (11, 36, 37). Factors contributing to underutilization or absence of PR services in Saudi Arabia include the lack of nationally accepted guidelines, insufficient provider awareness, low referral rates, inadequate transportation and hospital facilities, and a shortage of trained multidisciplinary rehabilitation teams (11, 20, 36, 37).

To better reduce the COPD burden, such as hospital admissions, cost, and mortality, PR should be actively applied and utilized through these suggested practical solutions:

- The PR should be effectively integrated into primary care clinics, such as respiratory clinics.
- Provide training for the primary care team, particularly family physicians and respiratory therapists, to fully understand the benefits and effectively deliver basic pulmonary rehabilitation programs.
- Tertiary hospitals and existing rehabilitation centers should have their own PR programs to ensure an even distribution across Saudi Arabia's regions.
- Implement tele-rehabilitation and digital health to deliver PR remotely and monitor patient progress and adherence, especially among patients in rural areas.
- Allocate funds to finance PR infrastructure, equipment, and workforce development.
- Integrate PR with comprehensive public health initiatives, including smoking cessation, air pollution control, and obesity management.

#### 4.3.2 Ambulatory/home oxygen access

While ambulatory and home oxygen therapy are part of Saudi Arabia's healthcare system, there is a lack of comprehensive national statistics on coverage, accessibility, and service delivery (20, 39). There are gaps in our knowledge of the full range of ambulatory and home oxygen treatment availability throughout the Kingdom, as the available information is mostly from regional studies and tertiary healthcare institutions, rather than nationwide surveillance (39, 40). Despite the proven benefits of long-term oxygen therapy (LTOT) for people living with COPD (41), significant barriers to accessing these services persist. First, gaps in the diagnostic pathway are characterized by low public awareness of COPD and limited PFTs, which are likely to reduce the number of appropriate referrals for oxygen therapy assessment (9–11). Second, the presence of geographic disparities in COPD care delivery across Saudi Arabia (20, 29). Third, the high number of specialized respiratory facilities in urban tertiary centers may pose access challenges for rural and remote populations in need of ambulatory and home oxygen support (39, 42). The existing evidence regarding ambulatory and home oxygen therapy indicates multiple essential research and development priorities for Saudi Arabia:

- Implementation of extensive data collection about home and ambulatory oxygen use, including patient demographics, equipment classifications, and geographical distribution trends.
- Investigate adherence to guidelines, prescribing patterns, and obstacles to the effective administration of oxygen treatment in various hospital environments.
- Conducting longitudinal research to explore clinical outcomes, quality of life, healthcare utilization, and adherence rates among Saudi patients using this kind of therapy.
- Exploring the health economy's effects and the performance of technology on the Saudi population to improve resource distribution and patient access.

**Figure 3:**
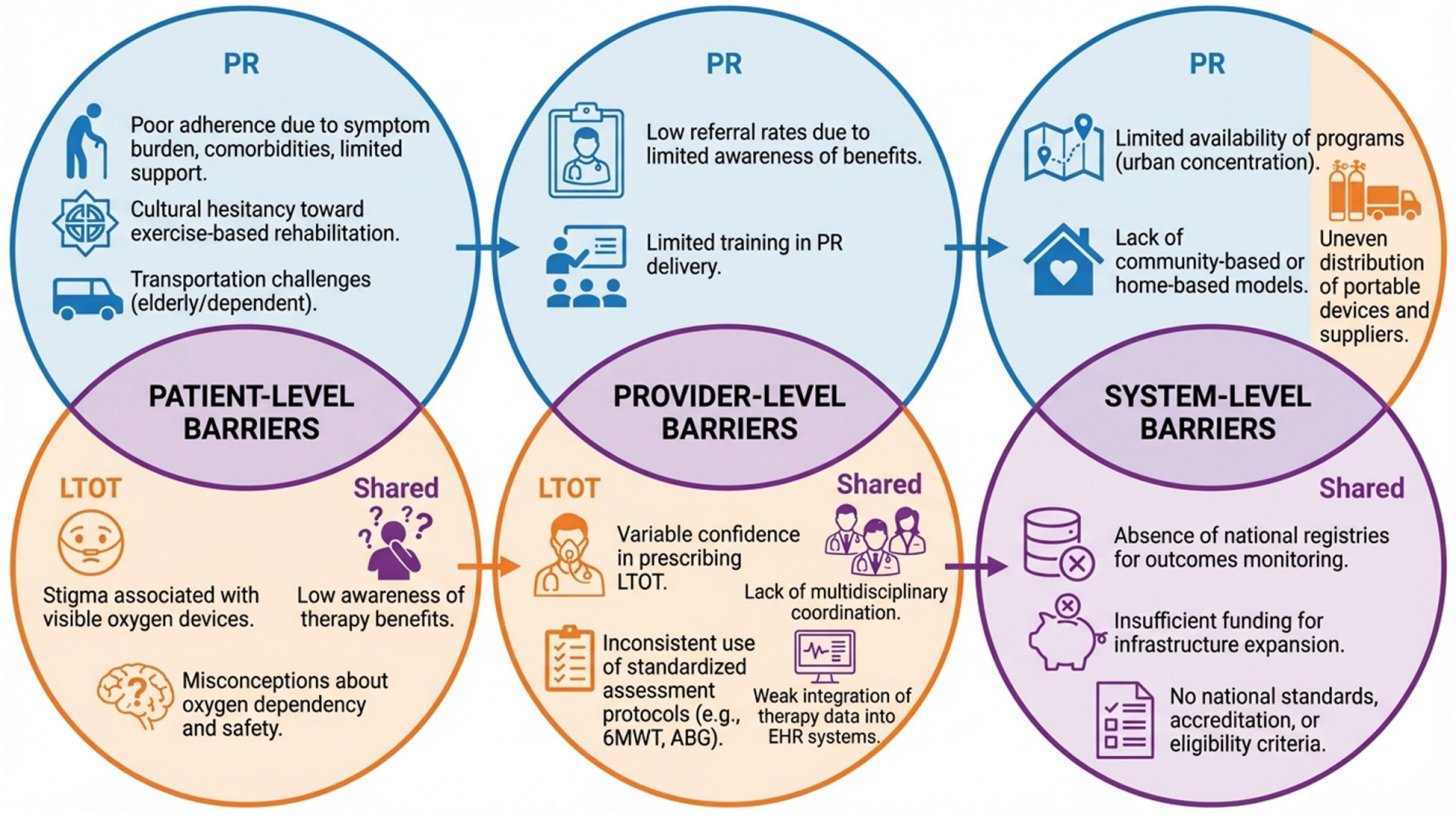
Main barriers for pulmonary rehabilitation and Ambulatory/home oxygen access in Saudi Arabia.

Saudi Arabia offers clinical standards and operational oxygen treatment facilities; however, data on ambulatory and home oxygen access are scarce. The national service coverage, reimbursement, availability of ambulatory devices, and patient outcomes remain unclear. Optimizing ambulatory and home oxygen therapy availability and outcomes for Saudi patients with COPD requires comprehensive research and monitoring to address these knowledge gaps. In summary, Figure 3 presents the main barriers for pulmonary rehabilitation and Ambulatory/home oxygen access in Saudi Arabia.

### 4.4 Availability of nicotine replacement therapy

As part of its Vision 2030, the Saudi Arabian government is committed to enhancing the quality of both preventive and therapeutic healthcare services. The government is working to reduce the number of infectious illnesses nationwide and promote early diagnosis and treatment. As per the WHO reports, the epidemic of tobacco use is recognized as a significant public health challenge globally, leading to over 7 million deaths annually, including over 1.5 million deaths resulting from non-smokers being exposed to second-hand smoke (43). Tobacco use and the associated risks to both users and second-hand smokers represent a significant public health issue in Saudi Arabia (44, 45). The use of tobacco products may result in a variety of health problems, which can lead to an increase in the burden and expense of healthcare in Saudi Arabia (7, 46, 47). According to statistics from the Saudi Ministry of Health, approximately 70,000 Saudis lose their lives each year due to diseases related to smoking (48). Further, it has been reported that in 2019, the age-standardized disability-adjusted life years (DALY) rate for COPD in Saudi Arabia was 508.15 per 100,000 population (95% UI 434.85–581.58). Additionally, COPD accounted for 1.65% (95% UI 1.39–1.88) of all deaths in the Kingdom that year and for 57% of deaths attributable to chronic respiratory diseases (7).

In response to the rising burden of smoking and its related diseases, the Saudi Arabian government has established robust efforts to combat tobacco that are in line with WHO's MPOWER measures (25). One of these efforts is the availability of tobacco dependence treatment (24, 25). The Saudi Guideline for Tobacco Cessation Services, established under national tobacco control policies, clearly advocates for pharmacological treatments, including nicotine replacement therapy (NRT), as an integral component of comprehensive cessation services, aligning with the principles of the WHO Framework Convention on Tobacco Control (49). Indeed, there is a national toll-free quitline that offers live help, and all of the primary drugs for quitting smoking, such as NRT, bupropion, and varenicline, are legally sold in the country as of December 2022 (25). Smokers can use NRT without a prescription, but other replacements need a prescription.

Furthermore, national health insurance fully covers all three alternatives, and NRT is among the medications listed as essential on the country's list of essential drugs (25). Support for smoking cessation is provided in various settings, including most primary care clinics, community services, hospitals, and professional offices. The provision of smoking cessation support is extensive across almost all regions of the country, with complete insurance coverage in place, except for community-based services, which remain uncovered (25). All in all, current evidence indicates that NRT is legally authorized in Saudi Arabia, widely available in community and online pharmacies, and routinely integrated into MOH and institutional smoking cessation initiatives, despite limited comprehensive national utilization data.

### 4.5 Digital health and COPD care in Saudi Arabia

The WHO and FDA define digital health as the use of digital technologies to enhance population health and accelerate progress toward healthcare for all (50, 51). Such technologies include various innovations such as mobile health, electronic health records, telemedicine, artificial intelligence, and data-based health platforms, all designed to enhance the accessibility, efficiency, and effectiveness of healthcare (50, 51). The integration of digital health is increasingly recognized as a vital element in the management of COPD care within Saudi Arabia, aligning with the objectives of the Vision 2030 health transformation agenda. While digital programs tailored for COPD are still in development, the establishment of national telehealth infrastructure, virtual call centers, and hospital-based telemedicine initiatives is laying the groundwork for a more organized approach to the remote management of chronic respiratory conditions.

Telemedicine has emerged as a fundamental element of the Saudi Digital Health Expansion, with Seha Virtual Hospital now recognized as the largest virtual care platform globally, linking more than 200 hospitals across the Kingdom. Virtual consultations, Tele-ICU services, and AI-driven diagnostics have become commonplace, particularly in remote areas. Such tools can be used for the diagnosis, management, and follow-up with people living with COPD. One example is the Internet of Medical Things (IoMT) for remote patient monitoring, where effective use can reduce hospital admissions and improve long-term outcomes for conditions such as COPD. According to the literature, the primary outcome measures associated with utilization of digital technologies in COPD included treatment adherence, health status, and quality of life (52). A national study conducted across Saudi Arabia on Virtual Home Health Care (VHHC) users found that patients with various chronic diseases regarded VHHC as effective in improving access, monitoring, and communication with healthcare providers (53). This supports the viability of implementing similar models for patients with COPD. Another effective digital tool that can be of great help to COPD patients is the national 937 virtual medical call center, which provides teleconsultations (54). Data collected from Saudi healthcare providers indicates that this tool enhances access and continuity of care (54). It can be utilized for triage, medication counseling, and providing exacerbation advice for patients with COPD. Research on the adoption of mobile health applications by Saudi physicians indicates increasing acceptance of mHealth tools for clinical decision support, communication, and chronic disease management (55). This trend suggests a favorable environment for implementing COPD-specific applications and digital pathways.

**Figure 4:**
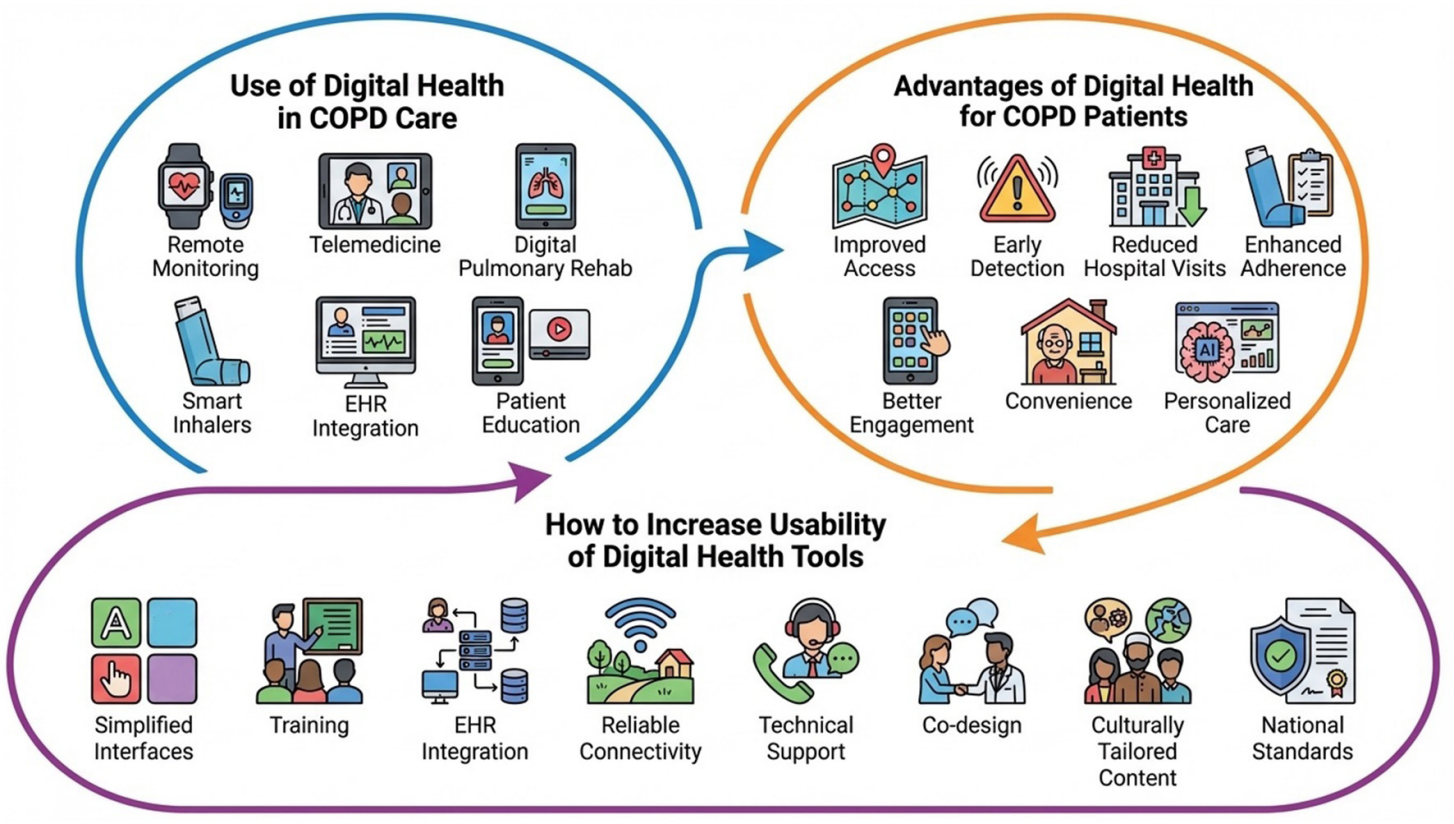
The key advantages and most effective strategies for increasing the usability of digital health tools in addressing COPD.

Nevertheless, implementing digital health presents several challenges. For instance, its application does not apply to all types of healthcare; implementation and adoption may be time-intensive and expensive, and extra caution is required when transferring patient health information (56, 57). The practical implementation of digital health for individuals living with COPD necessitates consideration of several key factors, including patient needs, technological familiarity, healthcare professional support, variable adherence, and data privacy (52, 58). The primary factors targeted for optimizing digital health in COPD across the Kingdom of Saudi Arabia should include patient engagement and determinants of user acceptance and adoption, including performance expectancy, effort expectancy, social influence, and facilitating conditions (55, 57, 59). Figure 4 highlights the key advantages and most effective strategies for improving the usability of digital health tools to address COPD.

Despite the widespread implementation of telehealth and digital platforms in Saudi Arabia, there remains a paucity of COPD-specific trials or registries that assess clinical outcomes, including exacerbation rates, emergency visits, and mortality associated with digital interventions (60, 61). Current data from Saudi Arabia highlight the need for culturally adapted digital programs for COPD that incorporate Arabic-language education, training on inhaler techniques, and remote monitoring within the national telehealth framework to optimize the effectiveness of digital health in managing COPD. Future work should investigate variations in digital health access across regions of the Kingdom (urban versus rural), as well as factors such as gender, age, and socioeconomic status, to prevent exacerbation of disparities in COPD care. Finally, future research should evaluate various COPD digital health models (telehealth, wearable devices, mHealth Apps, and artificial intelligence) in comparison to standard care within MOH and tertiary hospitals to assess cost-efficiency and adaptability.

## 5 Review limitations and strengths

The review was conducted narratively without following PRISMA standards, and reliance on a single author for study selection may have introduced selection bias. It also relies on some gray literature, government reports, and official statistics of variable quality, while possible language and database restrictions may have excluded relevant regional work. These limitations position the review as a synthesis that maps the COPD landscape and identifies priorities, rather than a quantitatively pooled estimate. Nevertheless, this review presents two distinct contributions to the existing literature. First, it offers an integrated policy-to-practice synthesis that connects the national regulatory and policy landscape with frontline access and care-coverage realities, rather than treating these domains separately. Second, it introduces the SCOPE framework as an original organizing structure that translates this synthesis into a coherent, actionable agenda for health-system transformation.

## 6 Conclusion

COPD is a rising respiratory and public health challenge in Saudi Arabia, and yet, most people living with this disease still face gaps in the care they receive. In many cases, COPD remains undiagnosed, the application of clinical guidelines is inconsistent, and access to pulmonary rehabilitation or coordinated community support is often limited. The fragmentation of digital and community-based services poses significant challenges for patients seeking continuous and coordinated care. Such challenges are exacerbated by an inadequate number of qualified respiratory specialists, irregular implementation of tobacco-control measures, and the absence of a robust national framework for monitoring disease trends and informing long-term strategies.

For healthcare transformation, Saudi Arabia must adopt an integrated national strategy that emphasizes early detection, enhances rehabilitation and home-based care, and strengthens digital integration across health services. Developing an effective respiratory care workforce and creating a strong data infrastructure will be crucial. Through the implementation of these reforms, the Kingdom is well positioned to advance toward a model of COPD care that prioritizes patient needs, promotes sustainability, aligns with international best practices, and improves health outcomes for individuals nationwide.

## Data Availability

The original contributions presented in the study are included in the article/supplementary material, further inquiries can be directed to the corresponding author.

## References

[1] Agustí A Celli BR Criner GJ Halpin DMG Anzueto A Barnes P . Global Initiative for Chronic Obstructive Lung Disease 2023 Report: GOLD Executive Summary. Eur Respir J. (2023) 61:2300239. doi: 10.1183/13993003.00239-2023 36858443 PMC10066569

[2] Boers E Barrett M Su JG Suri K Erqou S Bhatt SP . Global burden of chronic obstructive pulmonary disease through (2050). JAMA Netw Open. (2023) 6:e2346598-e2346598. doi: 10.1001/jamanetworkopen.2023.46598 PMC10704283 38060225

[3] Sin DD Anthonisen NR Soriano JB Agusti AG. Mortality in COPD: role of comorbidities. Eur Respir J. (2006) 28:1245. doi: 10.1183/09031936.00133805 17138679

[4] Alqahtani JS Njoku CM Bereznicki B Wimmer BC Peterson GM Kinsman L . Risk factors for all-cause hospital readmission following exacerbation of COPD: a systematic review and meta-analysis. Eur Respir Rev. (2020) 29:190166. doi: 10.1183/16000617.0166-2019 32499306 PMC9488450

[5] Chen S Kuhn M Prettner K Bloom DE. The global economic burden of chronic obstructive pulmonary disease for 204 countries and territories in 2020-50: a health-augmented macroeconomic modelling study. Lancet Glob Health. (2023) 11:e1183-e1193. doi: 10.1016/S2214-109X(23)00217-6 PMC10369014 37474226

[6] MEPs and respiratory health experts voice concern over budget cuts to lung health and warn of knock-on effects. (2024). Available online at: https://www.ersnet.org/news-and-features/news/meps-and-respiratory-health-experts-voice-concern-over-budget-cuts-to-lung-health-and-warn-of-knock-on-effects/ [accessed October 6, 2025]

[7] Alqahtani JS. Prevalence incidence morbidity and mortality rates of COPD in Saudi Arabia: Trends in burden of COPD from 1990 to (2019). PLoS ONE. (2022) 17:e0268772. doi: 10.1371/journal.pone.0268772 35588429 PMC9119447

[8] Wang H Naghavi M Allen C Barber RM Bhutta ZA Carter A . Global regional and national burden of chronic obstructive pulmonary disease from 1990 to (2019). Front Physiol. (2022) 13:925132. doi: 10.3389/fphys.2022.925132 36017339 PMC9396373

[9] Alqahtani JS Aldhahir AM Oyelade T Alghamdi SM Almehmadi M Alqahtani AS . A nationwide survey of public COPD knowledge and awareness in Saudi Arabia: A population-based survey of 15,000 adults. PLoS One. (2023) 18:e0287565. doi: 10.1371/journal.pone.0287565 37406018 PMC10321629

[10] Alobaidi NY Alharbi FF Alshammari TM Alqahtani JS. Awareness and barriers of adherence to chronic obstructive pulmonary disease guidelines among respiratory therapists. Ann Thorac Med. (2025) 20:108–16. doi: 10.4103/atm.atm_227_24 40236381 PMC11996134

[11] Aldhahir AM Alqahtani JS AlDraiwiesh IA Alghamdi SM Alsulayyim AS Alqarni AA . Healthcare providers' attitudes beliefs and barriers to pulmonary rehabilitation for patients with chronic obstructive pulmonary disease in Saudi Arabia: a cross-sectional study. BMJ Open. (2022) 12:e063900. doi: 10.1136/bmjopen-2022-063900 PMC9621177 36302583

[12] Speak Up for COPD – Health Policy Partnership. Available online at: https://speakupforcopd.com/wp-content/uploads/2024/10/The-global-state-of-COPD-driving-change-to-tackle-a-lung-health-crisis-4.pdf [accessed October 6, 2025]

[13] Adams EJ Taylor A Spencer A Taylor M Campbell JD Wilson MR. Estimating the Health and Economic Impact of Improved Management in Prevalent Chronic Obstructive Pulmonary Disease Populations in England Germany Canada and Japan: A Modelling Study. Int J Chron Obstruct Pulmon Dis. (2023) 18:2127–46. doi: 10.2147/COPD.S416988 37789931 PMC10543939

[14] Kiley JP Gibbons GH. COPD National Action Plan: Addressing a Public Health Need Together. Chest. (2017) 152:698–9. doi: 10.1016/j.chest.2017.08.1155 28991541

[15] Ghanei M Aslani J Peyman M Harandi AA Aarabi M Adibi P . National Plan for Chronic Respiratory Diseases Prevention and Control in Iran. Med J Islam Repub Iran. (2022) 36:170. doi: 10.47176/mjiri.36.170 37159756 PMC10163208

[16] Jones RCM Price D Chavannes NH Lee AJ Hyland ME Ställberg B . Summary of the consultation on a strategy for services for chronic obstructive pulmonary disease (COPD) in England. Prim Care Respir J. (2010) 19 Suppl 2:S1–s17. doi: 10.4104/pcrj.2010.00081 PMC6602277 21103802

[17] Stone PW Lowe D Pursey N Roberts CM. Comparison of COPD primary care in England Scotland Wales and Northern Ireland. NPJ Prim Care Respir Med. (2022) 32:46. doi: 10.1038/s41533-022-00305-8 36280669 PMC9592607

[18] Hurst JR Siddharthan T Contoli M D'Ambrosio CM Fawzy A Hossain MA. Challenges in the Implementation of Chronic Obstructive Pulmonary Disease Guidelines in Low- and Middle-Income Countries: An Official American Thoracic Society Workshop Report. Ann Am Thorac Soc. (2021) 18:1269–77. doi: 10.1513/AnnalsATS.202103-284ST 34328399 PMC8513652

[19] Khan JH Lababidi HM Al-Moamary MS Zeitouni MO Al-Jahdali HH Al-Amoudi OS . The Saudi Guidelines for the Diagnosis and Management of COPD. Ann Thorac Med. (2014) 9:55–76. doi: 10.4103/1817-1737.128843 24791168 PMC4005164

[20] Al-Jahdali H Alhamad EH Alqahtani JS Alghamdi SM Al Dabbagh R Mandal S . The Saudi Thoracic Society Evidence-based guidelines for the diagnosis and management of chronic obstructive pulmonary disease. Ann Thorac Med. (2025) 20:87–132. doi: 10.4103/atm.atm_155_24 39926399 PMC11804957

[21] Law of Management of Chemical Substances. (2006). Available online at: https://qanoniah.com/en/File/EdJG8m4pa1LVb5PLgr2bq5W60-Law-of-Management-of-Chemical-Substances-and-its-Executive-Regulation [accessed October 9, 2025]

[22] The GCC Unified Guidelines for Hazardous Chemical Substances Management (2021). Available online at: https://zoinet.org/wp-content/uploads/2023/02/GCC-guidelines_EN.pdf [accessed October 9, 2025]

[23] Masood MA Alqahtani JS Aldhahir AM Alghamdi SM Mandal S Hurst JR. Occupational health in the Gulf Cooperation Council (GCC): A systematic review and call for comprehensive policy development. PLoS One. (2024) 19:e0312251. doi: 10.1371/journal.pone.0312251 39656729 PMC11630603

[24] WHO. WHO report on the global tobacco epidemic 2023: Protect people from tobacco smoke. (2023). Available online at: https://www.who.int/publications/i/item/9789240077164 [accessed October 9, 2025]

[25] WHO. WHO report on the global tobacco epidemic 2023: Protect people from tobacco smoke: Country profile Saudi Arabia. (2023). Available online at: https://www.who.int/publications/m/item/tobacco-sau-2023-country-profile [accessed October 9, 2025]

[26] CHI Drug Formulary. Available online at: https://www.chi.gov.sa/en/Rules/Pages/DamanDrugFormulary.aspx#; https://www.chi.gov.sa/Style%20Library/IDF_Branding/Indication/140%20-%20Chronic%20Obstructive%20Pulmonary%20Disease%20(COPD)-Indication%20Update.pdf; https://www.chi.gov.sa/aboutchi/CCHIprograms/Documents/Tobacco%20.pdf [accessed October 26, 2025]

[27] ADDENDUM- November 2023 To the CHI Original Chronic Obstructive Pulmonary Disease Clinical Guidance- Issued January 2020. Available online at: https://www.chi.gov.sa/Style%20Library/IDF_Branding/Indication/140%20-%20Chronic%20Obstructive%20Pulmonary%20Disease%20(COPD)-Indication%20Update.pdf [accessed October 16, 2025]

[28] Naghavi M . Global burden of 292 causes of death in 204 countries and territories and 660 subnational locations 1990–2013: a systematic analysis for the Global Burden of Disease Study (2023) The Lancet. 10.1016/S0140-6736(25)01917-8 PMC12535838 41092928

[29] Tashkin DP Al Ahmari MD Al Dabbagh R Al Ghobain MO Alhamad EH Alqahtani JS . Prevalence and Management of Chronic Obstructive Pulmonary Disease in the Gulf Countries with a Focus on Inhaled Pharmacotherapy. J Aerosol Med Pulm Drug Deliv. (2024) 37:189–201. doi: 10.1089/jamp.2023.0016 38813999

[30] Perret JL Bonevski B McDonald VM Abramson MJ Marwick TH Toelle BG . Undiagnosed and ‘overdiagnosed'COPD using postbronchodilator spirometry in primary healthcare settings: a systematic review and meta-analysis. BMJ Open Respir Res. (2023) 10:e001478. doi: 10.1136/bmjresp-2022-001478 PMC10186431 37130651

[31] Al Ghobain MAM Al-Hajjaj MS Wali SO. Wali Prevalence of chronic obstructive pulmonary disease among smokers attending primary healthcare clinics in Saudi Arabia. Ann Saudi Med. (2011) 31:129–33. doi: 10.4103/0256-4947.77485 21403413 PMC3102470

[32] Márquez-Martín E Soriano JB Rubio MC Lopez-Campos JL. Differences in the use of spirometry between rural and urban primary care centers in Spain. Int J Chron Obstruct Pulmon Dis. (2015) 10:1633–9. doi: 10.2147/COPD.S86074 26316737 PMC4544627

[33] Alfaqeeh G Cook EJ Randhawa G Ali N. Access and utilisation of primary health care services comparing urban and rural areas of Riyadh Providence Kingdom of Saudi Arabia. BMC Health Serv Res. (2017) 17:106. doi: 10.1186/s12913-017-1983-z 28153002 PMC5288856

[34] Siraj RA Alqahtani JS Aldhahir AM Alghamdi SM Mandal S Hurst JR. Managing cognitive impairment in patients with chronic obstructive pulmonary disease (COPD) in Saudi Arabia: what are the current practices? Ann Med. (2025) 57:2413924. doi: 10.1080/07853890.2024.2413924 39876668 PMC11780691

[35] Siraj RA Alqahtani JS Aldhahir AM Alghamdi SM Al Dabbagh R Mandal S . Attitudes confidence barriers and current practice of managing depression in patients with COPD in Saudi Arabia: a national cross-sectional survey. BMJ Open. (2023) 13:e069670. doi: 10.1136/bmjopen-2022-069670 PMC10173993 37156583

[36] Aldhahir AM Alghamdi SM Alqahtani JS Al Dabbagh R Al Ahmari MD Alhamad EH . Pulmonary rehabilitation for COPD: A narrative review and call for further implementation in Saudi Arabia. Ann Thorac Med. (2021) 16:299–305. doi: 10.4103/atm.atm_639_20 34820017 PMC8588944

[37] Alahmadi FH. Optimizing Pulmonary Rehabilitation in Saudi Arabia: Current Practices Challenges and Future Directions. Medicina. (2025) 61:673. doi: 10.3390/medicina61040673 40282964 PMC12028724

[38] Ministry of Health at Saudi Arabia Annual Health Statistical Report (2023). Available online at: https://www.moh.gov.sa/en/Ministry/Statistics/book/Documents/Statistical-Yearbook-2023.pdf [accessed December 3, 2025]

[39] Alsubaiei ME Cafarella PA Frith PA McEvoy RD Effing TW. Current care services provided for patients with COPD in the Eastern province in Saudi Arabia: a descriptive study. Int J Chron Obstruct Pulmon Dis. (2015) 10:2379–91. doi: 10.2147/COPD.S89456 26604736 PMC4639520

[40] Beigh S Alghamdi SM Alqahtani JS Aldhahir AM Al Dabbagh R Mandal S . Management of Chronic Respiratory Diseases: A Retrospective Analysis of Demographic Predictors and Treatment Outcomes in Asthma and Chronic Obstructive Pulmonary Disease. J Nat Sci Med. (2025) 8:123–134. doi: 10.4103/jnsm.jnsm_164_24

[41] Croxton TL Bailey WC. Long-term oxygen treatment in chronic obstructive pulmonary disease: recommendations for future research: an NHLBI workshop report. Am J Respir Crit Care Med. (2006) 174:373–8. doi: 10.1164/rccm.200507-1161WS 16614349 PMC2648117

[42] Al-Otaibi HM. Characteristics and distribution of respiratory therapy practitioners in Saudi Arabia: national cross-sectional results. Hum Resour Health. (2024) 22:80. doi: 10.1186/s12960-024-00961-6 39604948 PMC11603939

[43] WHO. Tobacco Facts Sheet (2025). Available online at: https://www.who.int/news-room/fact-sheets/detail/tobacco [accessed December 23, 2025]

[44] Alasqah I Mahmud I East L Usher K. A systematic review of the prevalence and risk factors of smoking among Saudi adolescents Saudi *Medical J*. (2019) 40:867. doi: 10.15537/smj.2019.9.24477 PMC6790477 31522213

[45] Monshi SS Desouky EDE Aldukhail SK Al-Zalabani AH Alqahtani MM Dalatony MME . Tobacco use trends among youth in Saudi Arabia: 2007–(2022). Front Public Health. (2025) 13:1608394. doi: 10.3389/fpubh.2025.1608394 40672913 PMC12263556

[46] Alqahtani JS Aldhahir AM AlDraiwiesh IA Alamoudi AO Alqarni AA Alghamdi SM . Smoking prevalence among stroke patients in Saudi Arabia: A systematic review and meta-analysis. Heart & Lung. (2025) 74:142–51. doi: 10.1016/j.hrtlng.2025.07.005 40652614

[47] Alqahtani JS AlDraiwiesh IA Aldhahir AM Oyelade T. Burden of smoking-related stroke in Saudi Arabia: trends from 1990 to (2021). PLOS ONE. (2025) 20:e0324039. doi: 10.1371/journal.pone.0324039 40435184 PMC12118935

[48] Itumalla R Aldhmadi BK. Combating tobacco use in Saudi Arabia: a review of recent initiatives. East Mediterr Health J. (2020) 26:858–863. doi: 10.26719/emhj.20.019 32794172

[49] The Saudi Guideline for Tobacco Cessation Services. (2018). Available online at: https://www.chi.gov.sa/aboutchi/CCHIprograms/Documents/Tobacco%20.pdf [accessed December 23, 2025]

[50] WHO. Digital health. Available online at: https://www.who.int/health-topics/digital-health/#tab=tab_1 [accessed December 23, 2025]

[51] FDA. What is Digital Health? Available online at: https://www.fda.gov/medical-devices/digital-health-center-excellence/what-digital-health [accessed December 23, 2025]

[52] Alghamdi SM. Content Mechanism and Outcome of Effective Telehealth Solutions for Management of Chronic Obstructive Pulmonary Diseases: A Narrative Review. Healthcare. (2023) 11:3164. doi: 10.3390/healthcare11243164 38132054 PMC10742533

[53] Elsayed EA Aljehani NM Alanazi EM Sayed SH. Effectiveness of Virtual Home Health Care for Chronic Diseases Management: A Cross-Sectional Study Among the Saudi Population. J Multidiscip Healthc. (2025) 18:7025–33. doi: 10.2147/JMDH.S556449 41194906 PMC12584781

[54] Al-Wathinani AM Dhafar YO Aljarallah SA Alqahtani MS Alamri FA Aljohani AO . Healthcare Providers' Experience with Saudi Arabia's 937 Virtual Medical Call Centers and Telehealth. J Multidiscip Healthc. (2024) 17:2949–60. doi: 10.2147/JMDH.S467172 38933694 PMC11203774

[55] Alsahli S Hor SY. The adoption of mobile health applications by physicians during the COVID-19 pandemic in developing countries: The case of Saudi Arabia. Int J Inf Manag Data Insights. (2024) 4:100289. doi: 10.1016/j.jjimei.2024.100289

[56] Telehealth around the world. Available online at: https://www.dlapiperintelligence.com/telehealth/countries/?c=SA [accessed December 24, 2025]

[57] Alghamdi SM Aldhahir AM Alqahtani JS Siraj RA Alsulayyim AS Almojaibel AA . Healthcare Providers' Perception and Barriers Concerning the Use of Telehealth Applications in Saudi Arabia: A Cross-Sectional Study. Healthcare. (2022) 10:1527. doi: 10.3390/healthcare10081527 36011185 PMC9408269

[58] Althobiani MA Russell AM Jacob J Ranjan Y Ahmad R Folarin AA . The role of digital health in respiratory diseases management: a narrative review of recent literature. Front Med. (2025) 12:1361667. doi: 10.3389/fmed.2025.1361667 PMC11896871 40078397

[59] Alghamdi SM Rajah AMA Aldabayan YS Aldhahir AM Alqahtani JS Alzahrani AA. Chronic Obstructive Pulmonary Disease Patients' Acceptance in E-Health Clinical Trials. Int J Environ Res Public Health. (2021) 18:5230. doi: 10.3390/ijerph18105230 34069028 PMC8156037

[60] Alghamdi SM Alqahtani JS Aldhahir AM. Current status of telehealth in Saudi Arabia during COVID-19. J Family Community Med. (2020) 27:208–11. doi: 10.4103/jfcm.JFCM_295_20 33354152 PMC7745786

[61] Alghamdi SM Alsulayyim AS Alqahtani JS Aldhahir AM. Digital Health Platforms in Saudi Arabia: Determinants from the COVID-19 Pandemic Experience. Healthcare. (2021) 9:1517. doi: 10.20944/preprints202109.0393.v1 34828563 PMC8618772

